# Distributed coordinated attitude tracking control of a multi-spacecraft system with dynamic leader under communication delays

**DOI:** 10.1038/s41598-022-19367-2

**Published:** 2022-09-03

**Authors:** Zhanjie Zhou, Zhihao Zhang, Yan Wang

**Affiliations:** 1 Department of Control Science and Engineering, Harbin Institute of Technology, Harbin, 150001 People’s Republic of China; 2 School of Mechanical, Electrical and Information Engineering, Shandong University, Weihai, 264209 People’s Republic of China

**Keywords:** Engineering, Mathematics and computing

## Abstract

This paper is dedicated to the challenging issue of the cooperative attitude tracking control of a multi-spacecraft system under communication delays. A state estimator using the attitude information of the neighbors is designed for each follower spacecraft to estimate the time-varying attitude information of the leader spacecraft in the case that the leader spacecraft cannot directly communicate with all following spacecraft. By constructing auxiliary variables based on estimated values and proposing a fixed-time control law to ensure that the auxiliary variables of the spacecraft can reach zero in fixed time, which is independent of the initial state, the effects of time-varying reference attitudes on the system can be reduced. The attitude error and the estimation error are proven to converge to the region containing the origin by input-to-state stability theory combined with the Lyapunov–Krasovskii approach. To further illustrate the effectiveness of the proposed control algorithm, numerical simulation results are presented.

## Introduction

Multi-spacecraft distributed cooperative attitude control, an important branch of cooperative control, has received extensive attention in recent years. Compared with traditional centralized coordination methods, distributed cooperative attitude control has the advantages of high efficiency, strong robustness, and high reliability. Distributed attitude cooperative control is dedicated to synchronizing the attitude of each spacecraft to a common value. In Zhang and Demetriou^1^ and Ma et al.^2^, the leaderless cooperative control problem for multiple spacecraft systems was investigated. With this algorithm, the attitude of each spacecraft was synchronized; however, the attitude value of this synchronization was not set in advance, which means that the final spacecraft attitude was uncertain. Under the assumption that all followers obtained the leader’s acceleration, the coordinated tracking problem with a dynamic leader was investigated in Hong et al.^3^, and the proposed algorithm ensures that the attitude of each spacecraft can track the desired trajectory asymptotically. Du et al.^4^ studied the multi-spacecraft attitude tracking problem, and the proposed control algorithms could guarantee that each spacecraft tracks the desired time-varying attitude. Moreover, Zou et al.^5^ proposed a cooperative attitude control algorithm without velocity measurement.

The aforementioned literature on leader-follower multi-spacecraft guarantees only the asymptotic convergence of the spacecraft attitude error under the premise that all followers can obtain information from the leader spacecraft. Compared to asymptotic convergence, Liang et al.^6^ proposed a finite-time control scheme, which achieved finite-time convergence of spacecraft attitude error under arbitrary communication topologies. To improve the robustness of the system, Zou and Kumar^7^ studied the multi-spacecraft distributed attitude coordination control problem in the consideration of external disturbances, and the proposed control algorithms could guarantee that the system achieves finite-time stability. The existing literature on finite-time cooperative attitude control algorithms for multiple spacecraft systems has achieved fruitful results; however, the settling time of finite-time control algorithms is dependent on the initial conditions of the system. In Polyakov^8^, fixed-time convergence was proposed, which guarantees that the settling time is independent of the initial states; thus, the settling time of fixed-time algorithms is dependent on only the design parameters. Shi and Zhou^9^ studied the fixed-time attitude tracking problem of multi-spacecraft, and the algorithms could guarantee the convergence time of the system states within a fixed time. Following the idea exposed by Ton and Petersen^10^, a disturbance observer was employed in the fixed-time control algorithms to reduce the interference of the multi-spacecraft system. Similarly, taking the external interference and uncertainties of the system into account, the proposed algorithm guaranteed that the system states converge to the regions containing the origin in fixed time in Gao and Cai^11^.

In addition to the convergence rate of the system, the communication delay, which affects the control performance and even undermines the stability of the system, must be addressed for attitude tracking control. Researchers have made great efforts to study the communication delay problem in linear and nonlinear multiagent systems. Abdessameud and Tayebi^12^ proposed an attitude synchronization scheme that solves the leaderless problem under directed interconnection between rigid bodies with constant communication delays. Similarly, the frequency domain approach was employed to investigate the stability of linear systems under communication delays in Wang^13^.The Lyapunov–Krasovskii function method is an effective approach for nonlinear systems with time-varying delays. In the case that the reference attitude is time-varying and can be obtained by each spacecraft, the back-stepping technique and the Lyapunov–Krasovskii method contribute to the control law design in Guo et al.^14^. Zhou et al.^15^ proposed state feedback attitude synchronization tracking control algorithms and analyzed the delay-dependent stability of the delay system. However, the existing literature on attitude cooperative control for multiple spacecraft with time-varying communication delays focuses on leaderless synchronization or tracking with a dynamic leader under the assumption that the information of the leader is obtained by each follower.

Motivated by the above facts, a distributed attitude cooperative tracking control algorithm is proposed for a multi-spacecraft system with time-varying communication delays. Modeled by the Euler–Lagrange equation, the control objective is to ensure that each follower spacecraft attitude error converges to the region containing the origin when only a part of the follower spacecraft can acquire the dynamic attitude of the leader spacecraft. For the auxiliary variables constructed in this paper, a fixed-time control algorithm is added to make the convergence time of the auxiliary variables independent of the initial state, thereby reducing the influence of the time-varying reference state on the system.

Compared with existing works, the main contributions of this study are as follows. First, compared with Zhou et al.^15^ and Liang et al.^6^, not all follower spacecraft have access to the time-varying attitude information of the leader spacecraft under the directed communication topology. A distributed estimator is employed to estimate the attitude trajectory of the leader for each spacecraft using its neighbors’ information under communication delays. Second, compared with Zou and Kumar^7^ and Zhao and Jia^16^, in the controller design, a fixed-time control algorithm is combined so that the settling time of the auxiliary variable is independent of the initial state. This can reduce the impact of the time-varying reference target state on the overall system. Third, in contrast to Zhai and Xia^17^ in which the algorithm focused on the static leader case, the attitude tracking control algorithms with communication delays in this paper focus on tracking with a dynamic leader spacecraft.

**Notations**:$\otimes$ represents the Kronecker product. For a vector $x = {[{x_1}, \ldots ,{x_n}]^T}$, we use $\left\| x \right\|$ to denote the Euclidean norm. Define ${x^\alpha }\mathrm{{ = [}}{x_1}^\alpha ,\ldots ,{x_n}^\alpha {\mathrm{{]}}^T}$, ${[{\left| {{x_1}} \right| ^\alpha },\ldots ,{\left| {{x_n}} \right| ^\alpha }]^T}$, $si{g^\alpha }(x) = {[{\mathop{\rm sgn}} ({x_1}){\left| {{x_1}} \right|^\alpha },...,{\mathop{\rm sgn}} ({x_n}){\left| {{x_n}} \right|^\alpha }]^T}$ and for $x = {[{x_1},{x_2},{x_3}]^T}$, $${x^ \times } = \left[ {\begin{array}{*{20}{c}} 0&{}{ - {x_3}}&{}{{x_2}}\\ {{x_3}}&{}0&{}{ - {x_1}}\\ { - {x_2}}&{}{{x_1}}&{}0 \end{array}} \right]$$.

## Background

### Euler–Lagrange system

Consider a multiple rigid spacecraft system consisting of *n* followers, labeled as spacecraft $i,i = 1,\ldots ,n$, and one leader labeled as spacecraft 0. The attitude of the ith follower is described by modified Rodriguez parameters (MRPs) as

1 $$\begin{aligned} {\dot{\sigma }_i}\mathrm{{ = }}G({\sigma _i}){\omega _i}, \end{aligned}$$

where ${\sigma _i}\mathrm{{ = [}}{\sigma _{i1}},\mathrm{{ }}{\sigma _{i2}},\mathrm{{ }}{\sigma _{i3}}]\mathrm{{ }} \in {R^3}$ is the MRP of the *i*th spacecraft denoting the attitude orientation of the body-fixed frame with respect to the inertial frame. ${\omega _i} \in {R^3}$ is the angular velocity of the *i*th rigid body with respect to the inertial frame expressed in the body frame of the *i*th rigid body. $G({\sigma _i})$ is described as

2 $$\begin{aligned} G({\sigma _i})\mathrm{{ = }}\frac{1}{2}\left[ \frac{{1 - {{\left\| {{\sigma _i}} \right\| }^2}}}{2}{I_3} + {\sigma _i}^ \times + {\sigma _i}{\sigma _i}^T\right] . \end{aligned}$$

The matrix $G({\sigma _i})$ is provided with following properties

3 $$\begin{aligned}&\mathrm{{det(}}G({\sigma _i})) = \frac{{{{(1 + {\sigma _i}^2)}^3}}}{4} \ne 0 \nonumber \\&{G^T}({\sigma _i})G({\sigma _i})\mathrm{{ = (}}\frac{{1 + {\sigma _i}^2}}{4}{)^2}{I_3}. \end{aligned}$$

The dynamics of the *i*th spacecraft in the system are described by

4 $$\begin{aligned} {J_i}{\dot{\omega }_i} = - {\omega _i}^ \times {J_i}{\omega _i} + {u_i}, \end{aligned}$$

where $J \in {R^{3 \times 3}}$ is the inertia matrix of the *i*th spacecraft and ${u_i} \in {R^3}$ is the control torque of the *i*th spacecraft. The *n* followers are represented with Euler–Lagrange equations by combining (1) and (4) as

5 $$\begin{aligned} {M_i}({\sigma _i}){\ddot{\sigma }_i} + {C_i}({\sigma _i},{\dot{\sigma }_i}){\dot{\sigma }_i} = {\tau _i}, \end{aligned}$$

where ${C_i}({\sigma _i},{\dot{\sigma }_i}) = - {G^{ - T}}({\sigma _i}){J_i}{G^{ - 1}}({\sigma _i})\dot{G}({\sigma _i}){G^{ - 1}}({\sigma _i}) - {G^{ - T}}({\sigma _i}){({J_i}{\omega _i})^ \times }{G^{ - 1}}({\sigma _i})$, ${\tau _i}\mathrm{{ = }}{G^{ - T}}({\sigma _i}){u_i}$, and ${M_i}({\sigma _i}) = {G^{ - T}}({\sigma _i}){J_i}{G^{ - 1}}({\sigma _i})$. According to (3), ${M_i}({\sigma _i})$ is the symmetric positive-definite inertia matrix. For more details on the Euler–Lagrangian model, please refer to Meng and Ren^18^. Throughout the subsequent analysis, the following fundamental properties of system (5) are given by Zhai and Xia^17^:

**Property 1** Parameter Boundedness: For any *i*th spacecraft, there exist positive constants ${k_m}$, ${k_M}$ and ${k_C}$, $0 < {k_m}{I_3} \le {M_i}({\sigma _i}) \le {k_M}{I_3}$, $\left\| {{C_i}({\sigma _i},{{\dot{\sigma }}_i})} \right\| \le {k_C}\left\| {{{\dot{\sigma }}_i}} \right\|$.

**Property 2** Linearity in the dynamic parameters: ${M_i}({\sigma _i})x + {C_i}({\sigma _i},{\dot{\sigma }_i})y = {Y_i}({\sigma _i},{\dot{\sigma }_i},x,y){\Theta _i}$, for any $x,y \in {R^3}$, where ${\Theta _i}$ is the constant parameter vector associated with the *i*th spacecraft and ${Y_i}({\sigma _i},{\dot{\sigma }_i},x,y){\Theta _i}$ is a known regression matrix.

### Graph theory

Graph theory is briefly introduced to represent the topology of the information flow among multi-spacecraft system. A directed weighted graph can be denoted as $G = (N,E,A)$, where $N = ({n_0},{n_1}, \ldots ,{n_n})$ is a finite nonempty set of nodes and $E \subseteq N \times N$ is a set of unordered pairs of nodes. An edge $({n_i},{n_j}) \in E$ denotes that the *j*th spacecraft can obtain the information from the *i*th spacecraft and vice versa. The adjacency matrix $A = [{a_{ij}}] \in {R^{n \times n}}$ of graph *G* is defined such that adjacency elements ${a_{ij}}$ satisfy if $({n_i},{n_j}) \in E$, and ${a_{ij}}\mathrm{{ = 0}}$ otherwise. The Laplacian matrix $L = [{l_{ij}}] \in {R^{n \times n}}$ associated with *A* is defined as ${l_{ii}} = {\sum _{i \ne j}}{a_{ij}}$ and ${l_{ij}} = - {a_{ij}}$, where $i \ne j$. The directed graph *G* has a directed spanning tree if and only if there exists at least one node having a directed path to all other nodes. The leader adjacency matrix is defined as $B\mathrm{{ = }}diag({a_{10}},{a_{20}},\ldots ,{a_{n0}})$, where ${a_{i0}}\mathrm{{ = }}1$ if the *i*th spacecraft can communicate with the leader spacecraft and ${a_{i0}}\mathrm{{ = 0}}$ otherwise. Define $H = L + B$.

#### Assumption 1

The communication topology graph $\bar{G}$ of the multi-spacecraft system has a directed spanning tree.

#### Lemma 2.1

(Ren and Cao^19^) *If graph* $\bar{G}$ *has a directed spanning tree*, *all eigenvalues of matrix* *H* *have positive real parts*.

### Mathematic background

Consider the following system:

6 $$\begin{aligned} \dot{x} = g(t,x),x(0)\mathrm{{ = }}{x_0}, \end{aligned}$$

where $x \in {R^n}$ and $g:{R_ + } \times {R^n} \rightarrow {R^n}$ is a nonlinear function, which can be discontinuous. For system (6), the following definitions and lemmas are given.

#### Definition 1

(*Polyakov*^8^) The equilibrium of system (6) is fixed-time stable if it is finite-time stable, and the settling time $T({x_0})$ is uniformly bounded for any initial states, that is, $\exists {T_{\max }} > 0$, such that $T({x_0}) \le {T_{\max }}$, $\forall {x_0} \in {R^n}$.

#### Lemma 2.2

(Polyakov^8^) *If there exists a continuous radially unbounded function* $V:{R^n} \rightarrow {R_ + } \cup \{ 0\}$ *such that*

1. $$V(x) = 0 \Leftrightarrow x = 0;$$
2. *for any solution* *x*(*t*) *of* (6) *satisfies the inequality* ${D^*}V(x(t)) \le - {(\alpha {V^p}(x(t)) + \beta {V^q}(x(t)))^k}$ *for some* $\alpha ,\beta ,p,q,k> 0:pk < 1,qk > 1$, *then the origin is globally fixed-time stable for system* (6) *and the following estimate holds*:
  7 $$\begin{aligned} T({x_0}) \le \frac{1}{{{\alpha ^k}(1 - pk)}} + \frac{1}{{{\beta ^k}(qk - 1)}}\mathrm{{ }}\forall {x_0} \in {R^n}, \end{aligned}$$
  *where* $${D^*}f(t) = \mathop {\lim }\limits _{h \rightarrow 0 + } \sup \frac{{f(t + h) - f(t)}}{h}$$.

#### Lemma 2.3

(Khalil^20^) *Consider the system*

8 $$\begin{aligned} \dot{x} = f(t,x,u), \end{aligned}$$

*where* *f*(*t*, *x*, *u*) *is continuously differentiable and globally Lipschitz in* (*x*, *u*), *uniformly in t. If the unforced system* $\dot{x} = f(t,x,0)$ *has a globally exponentially stable equilibrium point at the origin* $x = 0$, *then the system* (8) *is input-to-state stable*.

#### Lemma 2.4

(Zuo and Lin^21^) *Let* ${\xi _1},{\xi _2},\ldots {\xi _n} \ge 0$. *Then*

9 $$\begin{aligned}&\sum \limits _{i = 1}^n {{\xi _i}^p \ge {{\left(\sum \limits _{i = 1}^n {{\xi _i}} \right)}^p}} ,if\mathrm{{ }}0 < p \le 1,\nonumber \\&\sum \limits _{i = 1}^n {{\xi _i}^p \ge {n^{1 - p}}{{\left(\sum \limits _{i = 1}^n {{\xi _i}} \right)}^p},if\mathrm{{ }}p} > 1. \end{aligned}$$

#### Lemma 2.5

(Wang et al.^22^) *For any scalar* ${\gamma _2}> {\gamma _1} > 0$, *symmetric positive definite matrix* $P \in {R^{m \times m}}$ *and vector function* $\varpi :[{\gamma _1},{\gamma _2}] \rightarrow {R^m}$ *such that the following integration holds*:

10 $$\begin{aligned} \int _{{\gamma _1}}^{{\gamma _2}} {{\varpi ^T}(\sigma )} P\varpi (\sigma )d\sigma \ge \frac{1}{{{\gamma _{12}}}}{[\int _{{\gamma _1}}^{{\gamma _2}} {\varpi (\sigma )} d\sigma ]^T}P[\int _{{\gamma _1}}^{{\gamma _2}} {\varpi (\sigma )} d\sigma ], \end{aligned}$$

*where* $${\gamma _{12}} = {\gamma _2} - {\gamma _1}$$.

#### Lemma 2.6

(Gahinet and Apkarian^23^) *The block matrix* $$\left[ {\begin{array}{*{20}{c}} {{A_1}}&{}B\\ {{B^T}}&{}{{A_2}} \end{array}} \right] \le 0$$ *if and only if* $$\left\{ \begin{array}{l} \mathrm{{ }}{A_1} \le 0\\ {A_2} - {B^T}{A_1}^{ - 1}B \le 0 \end{array} \right.$$.

## Coordinated attitude tracking control with a dynamic leader under communication delays

In this section, we address the distributed coordinated attitude tracking control problem for multi-spacecraft under directed communication graph with a dynamic leader under communication delays. The attitude of the leader spacecraft is generated as^24^

11 $$\begin{aligned} \dot{v}&= Qv\nonumber \\ {\sigma _0}&= Nv, \end{aligned}$$

where $Q \in {R^{m \times m}}$ and $N \in {R^{3 \times m}}$ are constant matrices and $v \in {R^m}$ is an auxiliary variable.

### Remark 1

System (11) can generate multiple types of ${\sigma _0}$, such as step signals of arbitrary amplitude, ramp signals of arbitrary slope, and sinusoidal signals of arbitrary amplitudes and initial phases. By the reasonable design of *N*, a linear combination of *v* will generate a variety of different signals^25^. We assume $m = 3$ in this paper.

Define the attitude tracking error and its derivative of each follower spacecraft as

12 $$\begin{aligned} {\tilde{\sigma }_i}&= {\sigma _i} - {\sigma _0}\nonumber \\ {\dot{\tilde{\sigma _i}}}&= {\dot{\sigma }_i} - {\dot{\sigma }_0}, \end{aligned}$$

where $i = 1,2\ldots ,n$, and the control objective of this paper is to ensure $\mathop {\lim }\limits _{t \rightarrow \infty } {\tilde{\sigma }_i}(t) = 0$, $\mathop {\lim }\limits _{t \rightarrow \infty } {\dot{\tilde{\sigma _i}}}(t) = 0$. Because of the existence of time delay in communication between spacecraft, the distributed estimator is proposed as

13 $$\begin{aligned} {\dot{\hat{v_i}}}(t)\mathrm{{ = }}Q{\hat{v}_i}(t) - \alpha \sum \limits _{j = 0}^n {{a_{ij}}} [{\hat{v}_i}(t - T) - {\hat{v}_j}(t - T)], \end{aligned}$$

where ${\hat{v}_i}(t)$ is the estimate of attitude information ${v_0}(t)$, $\alpha$ is a positive constant and. *T* is the time-varying communication delay.

### Remark 2

In this paper, the communication time delay between the available spacecraft is considered. There is ${\hat{v}_i}(t - T)$ in (13), however, this item is not caused by the communication time delay, but the states of the *i*th spacecraft at the time $t - T$ is called during the controller calculation process.

### Assumption 2

The time-varying communication delay *T* satisfies $0 \le T \le {T_0}$ and $0 \le \dot{T}(t) \le d < 1$, where ${T_0}$ is a positive constant and there exist positive definite matrices ${P_1}$, ${P_2}$, *W* and reasonably matrices *Z* such that

$$\begin{aligned} A = \left[ {\begin{array}{*{20}{c}} {{P_1} + 2\mathrm{{(}}Q \otimes {I_n}) + dW + d{{\mathrm{{(}}Q \otimes {I_n})}^T}{P_2}\mathrm{{(}}Q \otimes {I_n}) + 2Z}&{}{ - \alpha [H \otimes {I_3} + d{{\mathrm{{(}}Q \otimes {I_n})}^T}{P_2}\mathrm{{(}}H \otimes {I_3})\mathrm{{]}} - Z\mathrm{{]}}}\\ { - \alpha [H \otimes {I_3} + d{{\mathrm{{(}}Q \otimes {I_n})}^T}{P_2}\mathrm{{(}}H \otimes {I_3})\mathrm{{]}} - Z{\mathrm{{]}}^T}}&{}{ - (1 - d){P_1} + d{\alpha ^2}{{\mathrm{{(}}H \otimes {I_3})}^T}{P_2}\mathrm{{(}}H \otimes {I_3})} \end{array}} \right] \le 0. \end{aligned}$$

### Remark 3

According to Lemma 2.6, let $$A = \left[ {\begin{array}{*{20}{c}} {{A_{11}}}&{}{{A_{12}}}\\ {{A_{12}}^T}&{}{{A_{22}}} \end{array}} \right]$$, it is feasible to make matrix ${A_{11}}$ a negative definite matrix by choosing matrix *Z* reasonably. The positive definite ${P_1}$, ${P_2}$ and *H* could make matrix ${A_{22}}$ a negative definite matrix; thus, ${A_{22}} - {A_{12}}^T{A_{11}}^{ - 1}{A_{12}} \le 0$ is feasible. For example, we can choose a negative definite matrix *Z* reasonably. Compared with Wang et al.^26^, although the LMI forms are similar, the design of this paper can guarantee each block matrix on main diagonal negative definite, thus guaranteeing the correctness of the proof. We first give the boundedness proof for the relevant states (13). Consider the following Lyapunov–Krasovskii functional

14 $$\begin{aligned} \begin{array}{l} {V_1} = {{\hat{v}}^T}(t)\hat{v}(t) + \int _{t - T}^t {{{\hat{v}}^T}(\zeta )} {P_1}\hat{v}(\zeta )d\zeta + \int \limits _{ - T}^o {\int \limits _{t + \theta }^t {{{\dot{\hat{v}}}^T}(\zeta ){P_2}\dot{ \hat{v}}(\zeta )d\zeta } } d\theta \\ \,\,\,\,\,\,\,\,\,\,\mathrm{{ }} + \int \limits _0^t {\int \limits _{\theta - T}^\theta {\left[ {\begin{array}{*{20}{c}} {{v^T}(\theta )}&{}{{{\dot{\hat{v}}}^T}(\zeta )} \end{array}} \right] \left[ {\begin{array}{*{20}{c}} W&{}{{Z^T}}\\ Z&{}{{P_2}} \end{array}} \right] \left[ {\begin{array}{*{20}{c}} {\hat{v}(\theta )}\\ {\dot{\hat{v}}(\zeta )} \end{array}} \right] d\zeta } } d\theta , \end{array} \end{aligned}$$

where $\hat{v}(t)$ is the column vector of column ${\hat{v}_i}(t)$, and the derivative of ${V_1}$ is

15 $$\begin{aligned} \begin{array}{l} {{\dot{V}}_1}\mathrm{{ = }}2{{\hat{v}}^T}(t)\dot{ \hat{v}}(t) + \hat{v}(t){P_1}\hat{v}(t) - (1 - \mathop T\limits ^. ){{\hat{v}}^T}(t - T){P_1}\hat{v}(t - T) - \int \limits _{t - T}^t {{{\dot{ \hat{v}}}^T}(\zeta ){P_2}\dot{ \hat{v}}(\zeta )d\zeta } \\ \,\,\,\,\,\,\,\,\,\,\mathrm{{ + }}T\dot{ \hat{v}}(t){P_2}\dot{ \hat{v}}(t)\mathrm{{ + }}T{{\hat{v}}^T}(t)W\hat{v}(t)\mathrm{{ + }}2{{\hat{v}}^T}(t)Z\left[ {\hat{v}(t) - \hat{v}(t - T)} \right] + \int \limits _{t - T}^t {{{\dot{ \hat{v}}}^T}(\zeta ){P_2}\dot{ \hat{v}}(\zeta )d\zeta } \\ \,\,\,\,\,\mathrm{{ }} \le {{\hat{v}}^T}(t)[ - 2\alpha (H \otimes {I_3}) - 2\alpha d{\mathrm{{(}}Q \otimes {I_n})^T}{P_2}\mathrm{{(}}H \otimes {I_3}) - 2Z\mathrm{{]}}\hat{v}(t - T) + \hat{v}(t)[{P_1} + 2\mathrm{{(}}Q \otimes {I_n})\\ \,\,\,\,\,\,\,\,\mathrm{{ }} + dW + d{\mathrm{{(}}Q \otimes {I_n})^T}{P_2}\mathrm{{(}}Q \otimes {I_n}) + 2Z\mathrm{{]}}\hat{v}(t) - {{\hat{v}}^T}(t - T)\mathrm{{[}}(1 - d){P_1} - d{\alpha ^2}{\mathrm{{(}}H \otimes {I_3})^T}{P_2}\mathrm{{(}}H \otimes {I_3})\mathrm{{]}}\hat{v}(t -T)\\ \,\,\,\,\,\mathrm{{ = [}}{{\hat{v}}^T}(t)\mathrm{{ }}{{\hat{v}}^T}(t - T)\mathrm{{ ]}}A{\mathrm{{[}}{{\hat{v}}^T}(t)\mathrm{{ }}{{\hat{v}}^T}(t - T)\mathrm{{ ]}}^T}\le 0 \end{array} \end{aligned}$$

therefore, $\hat{v}(t)$ is bounded according to Assumption 2 and (14).

Define auxiliary variables as

16 $$\begin{aligned}&{\dot{\sigma }_{ri}}(t) = NQ{\hat{v}_i}(t) - \beta \sum \limits _{j = 0}^n {{a_{ij}}} [{\sigma _i}(t - T) - {\sigma _j}(t - T)] \end{aligned}$$

17 $$\begin{aligned}&{s_i}(t) = {\dot{\sigma } _i}(t) - {\dot{\sigma }_{ri}}(t), \end{aligned}$$

where $\beta$ is a positive constant. We obtain by Property 2,

18 $$\begin{aligned} {M_i}({\sigma _i}){\ddot{\sigma }_{ri}} + {C_i}({\sigma _i},{\dot{\sigma }_i}){\dot{\sigma }_{ri}} = {Y_i}({\sigma _i},{\dot{\sigma }_i},{\ddot{\sigma }_{ri}},{\dot{\sigma }_{ri}}){\Theta _i}. \end{aligned}$$

Combining (5) and (18), the coordinated attitude tracking control law for the *i*th follower spacecraft is proposed as

19 $$\begin{aligned} {\tau _i} = {C_i}({\sigma _i},{\dot{\sigma }_i}){s_i} + {Y_i}{\Theta _i} - {k_1}{M_i}({\sigma _i})si{g^p}({s_i}) - {k_2}{M_i}({\sigma _i})si{g^q}({s_i}) - {k_3}{s_i}, \end{aligned}$$

where ${k_1}$ and ${k_2}$ are positive constants, $0< p < 1$ and $q > 1$.

### Theorem 1

*Considering the multi-spacecraft attitude system* (5), *the auxiliary variable* ${s_i}$ *of each follower spacecraft converges to zero in fixed time by properly designing the parameter coordinated control law* (19).

### Proof

We have shown that the states $\hat{v}$ are bounded in (14); moreover, ${\dot{\hat{v}}_i}$, ${\dot{\sigma }_{ri}}$ and ${\ddot{\sigma }_{ri}}$ are bounded from (13) and (16). By Property 1, ${Y_i}({\sigma _i},{\dot{\sigma }_i},{\ddot{\sigma }_{ri}},{\dot{\sigma }_{ri}}){\Theta _i}$ is bounded for bounded states ${\ddot{\sigma }_{ri}}$ and ${\dot{\sigma }_{ri}}$. $\tau$, *s*and $\Theta$ are column stack vectors of ${\tau _i}$, ${s _i}$ and ${\Theta _i}$,respectively. $C(\sigma ,\dot{\sigma })$, $M(\sigma )$and *Y* are block diagonal matrices of ${C_i}({\sigma _i},{\dot{\sigma }_i})$, ${M_i}({\sigma _i})$ and ${Y_i}$, respectively. Substituting (17), (18) and (19) into (5), the system can be expressed as

20 $$\begin{aligned} M(\sigma )\dot{s} = - {k_1}M(\sigma )si{g^p}(s) - {k_2}M(\sigma )si{g^q}(s) - {k_3}s. \end{aligned}$$

Consider the Lyapunov function candidate as

21 $$\begin{aligned} {V_2}\mathrm{{ = }}\frac{1}{2}{s^T}s. \end{aligned}$$

Taking the time derivative of the Lyapunov function ${V_2}$, by means of Lemma 2.4, we obtain

22 $$\begin{aligned} \begin{array}{l} {{\dot{V}}_2} = {s^T}\dot{s}\\ \,\,\,\,\,\,\,\,= - {s^T}{M^{ - 1}}(\sigma )({k_1}M(\sigma )si{g^p}(s) + {k_2}M(\sigma )si{g^q}(s) + {k_3}s)\mathrm{{ }}\\ \,\,\,\,\,\,\,\,= - {k_1}{s^T}si{g^p}(s) - {k_2}{s^T}si{g^q}(s) - {k_3}{s^T}{M^{ - 1}}(\sigma )s\\ \,\,\,\,\,\,\mathrm{{ }} \le - {k_1}\sum \limits _{i = 1}^n {\sum \limits _{I = 1}^3 {{{({s_{iI}}^2)}^{\frac{{p + 1}}{2}}}} } - {k_2}\sum \limits _{i = 1}^n {\sum \limits _{I = 1}^3 {{{({s_{iI}}^2)}^{\frac{{q + 1}}{2}}}} } \\ \,\,\,\,\,\,\mathrm{{ }} \le - {k_1}[\sum \limits _{i = 1}^n {\sum \limits _{I = 1}^3 {({s_{iI}}^2){]^{\frac{{p + 1}}{2}}}} } - {k_2}{(3n)^{\frac{{1 - q}}{2}}}[\sum \limits _{i = 1}^n {\sum \limits _{I = 1}^3 {({s_{iI}}^2){]^{\frac{{q + 1}}{2}}}} } \\ \,\,\,\,\,\,\mathrm{{ }} \le - {k_1} \cdot {2^{\frac{{p + 1}}{2}}}{V_2}^{\frac{{p + 1}}{2}} - {(3n)^{\frac{{1 - q}}{2}}}{k_2} \cdot {2^{\frac{{q + 1}}{2}}}{V_2}^{\frac{{q + 1}}{2}}. \end{array} \end{aligned}$$

Since $M(\sigma )$ is positive definite, we can obtain ${\dot{V}_2} < 0$, which means that ${s_i}$ is bounded. According to the Property 1 and Lemma 2.2, the auxiliary variable ${s_i}$ for the *i*th spacecraft will converge to zero in fixed time with a settling time

23 $$\begin{aligned} {T_s} \le \frac{{{2^{\frac{{1 - p}}{2}}}}}{{{k_1}(1 - p)}} + \frac{{{{(\frac{2}{{3n}})}^{\frac{{1 - q}}{2}}}}}{{{k_2}(q - 1)}}. \end{aligned}$$

The proof of Theorem 1 is complete. $\square$

Define ${\tilde{v}_i}(t) = {\hat{v}_i}(t) - v(t)$. $\tilde{\sigma }(t)$ and $\tilde{v}(t)$ are the column stack vectors of ${\tilde{\sigma }_i}(t)$ and ${\tilde{v}_i}(t)$, respectively. Rewrite (13) and (16) as

24 $$\begin{aligned}&\dot{\tilde{v}}(t)\mathrm{{ = (}}Q \otimes {I_n})\tilde{v}(t) - \alpha (H \otimes {I_3})\tilde{v}(t - T), \end{aligned}$$

25 $$\begin{aligned}&\dot{\tilde{\sigma }} (t)\mathrm{{ = }}(NQ \otimes {I_n})\tilde{v}(t) - \beta (H \otimes {I_3})\tilde{\sigma }(t - T) + s. \end{aligned}$$

Consider the following system

26 $$\begin{aligned} \left\{ \begin{array}{l} \dot{\tilde{\sigma }} (t)\mathrm{{ = }}(NQ \otimes {I_n})\tilde{v}(t) - \beta (H \otimes {I_3})\tilde{\sigma }(t - T)\\ \dot{\tilde{v}}(t)\mathrm{{ = (}}Q \otimes {I_n})\tilde{v}(t) - \alpha (H \otimes {I_3})\tilde{v}(t - T) \end{array} \right. \end{aligned}$$

define $$R=\left[ {\begin{array}{*{20}{c}} {\beta (H \otimes {I_3})}&{}\mathrm{{0}}\\ \mathrm{{0}}&{}{\alpha (H \otimes {I_3})} \end{array}} \right]$$, $$E = \left[ {\begin{array}{*{20}{c}} 0&{}{NQ \otimes {I_n}}\\ 0&{}{Q \otimes {I_n}} \end{array}} \right]$$, $F = \alpha (H \otimes {I_3}) - Q \otimes {I_n}$ and $U = R - E$. Let $x = {\left[ {{{\tilde{\sigma }}^T},{{\tilde{v}}^T}} \right] ^T}$. The system can be written in the following form

27 $$\begin{aligned} \dot{x}(t) = Ex(t) - Rx(t - T). \end{aligned}$$

### Remark 4

According to Lemma 2.1, all eigenvalues of matrix *H* have positive real parts. In the case of the establishment of Assumption 1, matrix *F* is positive definite by reasonably selecting positive constant $\alpha$. Thus, the block matrix *U* can be a positive definite matrix.

### Theorem 2

*Consider the linear system with communication delays described by* (27). *If there exist positive definite matrices* ${P_3}$, ${P_4}$ and ${P_5}$ *such that the following linear matrix inequality (LMI) holds*:

28 $$\begin{aligned} B = \left[ {\begin{array}{*{20}{c}} { - 2{P_3}U + d{P_4}\mathrm{{ }}}&{}{{P_3}R + (1 - d){P_4}}\\ {{R^T}{P_2} + (1 - d){P_4}\mathrm{{ }}}&{}{ - {P_5} - (1 - d){P_4}} \end{array}} \right] + {T_0}^2\left[ {\begin{array}{*{20}{c}} { - {U^T}}\\ {{R^T}} \end{array}} \right] {R^T}\left[ {\begin{array}{*{20}{c}} { - U}&R \end{array}} \right] < 0 \end{aligned}$$

*then system state* *x* *converges to zero asymptotically*. *Under Assumptions* 1*and* 2, *the distributed estimator* (13) *and the coordinated control law* (19), *which depends on* (16) *and* (17), *can guarantee that the attitude tracking error of the follower spacecraft asymptotically converges to zero*.

### Remark 5

The form of LMI (28) is inspired by (Polyak et al.^27^). Similar to the analysis in Remark 3, the corresponding solutions ${P_3}$, ${P_4}$ and ${P_5}$ can be obtained by the mature solution method of linear matrix inequality (Last^28^). In LMI (28), the upper bound ${T_0}$ of the communication delay that the system can cope with only related to design parameters and not to the structure of the controller itself. Therefore, ${T_0}$ does not affect the proof of system stability. But in practice, the size of ${T_0}$ affects the selection of ${\alpha }$ and ${\beta }$.

### Proof

Consider the following Lyapunov–Krasovskii functional for system (27):

29 $$\begin{aligned} {V_3} = {x^T}(t){P_3}x(t) + \int _{t - T}^t {{x^T}(\zeta ){P_4}x(\zeta )d\zeta } + T\int \limits _{ - T}^o {\int \limits _{t + \theta }^t {{{\dot{x}}^T}(\zeta ){P_5}\dot{x}(\zeta )d\zeta } } d\theta . \end{aligned}$$

Taking the time derivative of the Lyapunov–Krasovskii function ${V_3}$, we have

30 $$\begin{aligned} \begin{array}{l} {{\dot{V}}_3} = 2{x^T}(t){P_3}\dot{x}(t) + {x^T}(t){P_4}x(t) - (1 - \dot{T}){x^T}(t - T){P_4}x(t - T) + {T^2}{{\dot{x}}^T}(t){P_5}\dot{x}(t)\\ - T\int _{t - T}^t {{{\dot{x}}^T}(\zeta ){P_5}\dot{x}(\zeta )} d\zeta . \end{array} \end{aligned}$$

By employing the Leibniz–Newton formula, we obtain $\int _{t - T}^t {\dot{x}(\zeta )d} \zeta = x(t) - x(t - T)$. Define $\bar{x}(t) = x(t) - x(t - T)$. It can be obtained that the following inequality holds according to Lemma 2.5

31 $$\begin{aligned} {\bar{x}^T}(t){P_5}\bar{x}(t) \le T\int _{t - T}^t {{{\dot{x}}^T}(\zeta ){P_5}\dot{x}(\zeta )} d\zeta . \end{aligned}$$

Under Assumption 2, the following inequality can be obtained by using (31):

32 $$\begin{aligned}&{{\dot{V}}_3} \le 2x{(t)^T}{P_3}[Ex(t) - Rx(t - T)] + {x^T}(t){P_4}x(t) - (1 - \dot{T}){x^T}(t - T){P_4}x(t - T) + {T_0}^2{{\dot{x}}^T}(t){P_5}\dot{x}(t)\nonumber \\&\quad \quad - {{\bar{x}}^T}(t){P_5}\bar{x}(t)\nonumber \\&\quad \le 2{x^T}(t){P_3}[(E - R)x(t) + R\bar{x}(t)] + {x^T}(t){P_4}x(t) - (1 - d){(x(t) - \bar{x}(t))^T}{P_4}(x(t) - \bar{x}(t))\nonumber \\&\quad \quad + {T_0}^2{[(E - R)x(t) + R\bar{x}(t)]^T}{P_5}[(E - R)x(t) + R\bar{x}(t)] - {{\bar{x}}^T}(t){P_5}\bar{x}(t)\nonumber \\&\quad \le {x^T}(t)[ - 2{P_3}U + d{P_4}]x(t) + 2{x^T}(t)[{P_3}R + (1 - d){P_4}]\bar{x}(t) - {{\bar{x}}^T}(t)[{P_5} + (1 - d){P_4}]\bar{x}(t)\nonumber \\&\quad \quad + {T_0}^2{[ - Ux(t) + R\bar{x}(t)]^T}{P_5}[ - Ux(t) + R\bar{x}(t)]\nonumber \\&\quad = \left[ {{x^T}(t)}{\mathrm{{ }}\bar{x}{{(t)}^T}} \right] B{\left[ {{x^T}(t)} {\mathrm{{ }}\bar{x}{{(t)}^T}} \right] ^T} < 0. \end{aligned}$$

By substituting (28) into (32), linear system (27) is asymptotically stable, which means that $\mathop {\lim }\limits _{t \rightarrow \infty } \tilde{\sigma }(t) = 0$ and $\mathop {\lim }\limits _{t \rightarrow \infty } \tilde{v}(t) = 0$. Since the linear system (27) is asymptotically convergent, its characteristic roots all have negative real parts, so it is exponentially convergent. It thus follows that when $s = 0$, the system given by (24) and (25) is globally exponentially stable at the origin ${\left[ {{{\tilde{\sigma }}^T},{{\tilde{v}}^T}} \right] ^T} = 0$. Combining that *s*(*t*) has been proven to be bounded and converge to zero in fixed time in Theorem 1, the system given by (24) and (25) is input-to-state stable with respect to the input *s*(*t*) from Lemma 2.3. From Theorem 1, ${\dot{V}_2} \rightarrow 0$ as $t \rightarrow {T_s}$,i.e., $s(t) \rightarrow 0$ as $t \rightarrow {T_s}$. Since systems (24) and (25) are input-to-state stable with respect to the input *s*(*t*) and the state ${\left[ {{{\tilde{\sigma }(t)}^T},{{\tilde{v}}(t)^T}} \right] ^T}$, ${\dot{V}_3} \rightarrow 0$ as $t \rightarrow \infty$, i.e., $\tilde{\sigma }(t) \rightarrow 0$ and $\tilde{v}(t) \rightarrow 0$ as $t \rightarrow \infty$. This completes the proof of Theorem 2. $\square$

## Simulation results

**Figure 1:**
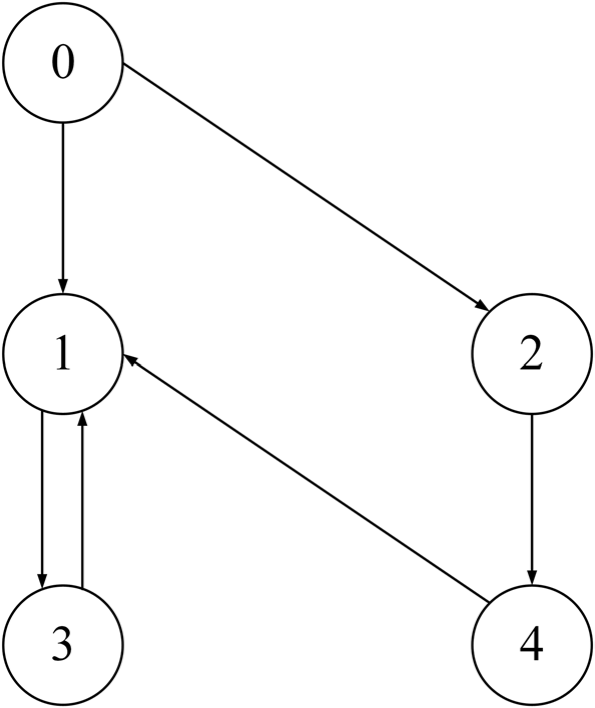
Communication topology.

In this section, to demonstrate the validity of the proposed control algorithms, numerical simulations for a multi-spacecraft system are conducted, and the communication topology is described by Fig. 1. The system consisting of four follower spacecraft and one leader spacecraft satisfies Assumption 1. Table 1 lists the inertia matrix and initial conditions of the follower spacecraft. The time-varying communication delay *T* is set as $T = 0.1 + 0.1\sin (t)$, which satisfies Assumption 2. The parameters of the distributed estimator (13), the auxiliary variable ${\dot{\sigma }_{ri}}$ and the controller (19) are set as $\alpha \mathrm{{ = }}1$, $\beta = 1$, ${k_1} = 0.8$, ${k_2} = 1$, ${k_3} = 1$, $p = 0.4$ and $q = 2$, respectively. The attitude ${\sigma _0}$ of the leader is generated by the following parameters:$$v(t) = \left[ \begin{array}{l} - 0.005 \times \sin (0.1t)\\ 0.008 \times \cos (0.1t)\\ - 0.007 \times \sin (0.1t) \end{array} \right]$$,$$Q = \left[ {\begin{array}{*{20}{c}} {0\mathrm{{ }}}&{}{ - \frac{1}{{16}}\mathrm{{ }}}&{}{0\mathrm{{ }}}\\ {\frac{1}{{50}}}&{}{0\mathrm{{ }}}&{}{\frac{1}{{10}}}\\ {\frac{1}{5}}&{}{ - \frac{7}{{80}}\mathrm{{ }}}&{}{ - \frac{1}{7}} \end{array}} \right]$$,$$N = \left[ {\begin{array}{*{20}{c}} { - 2}&{}\mathrm{{0}}&{}\mathrm{{0}}\\ \mathrm{{0}}&{}{\mathrm{{1}}\mathrm{{.6}}}&{}\mathrm{{0}}\\ \mathrm{{0}}&{}\mathrm{{0}}&{}{ - 2} \end{array}} \right]$$.

**Figure 2:**
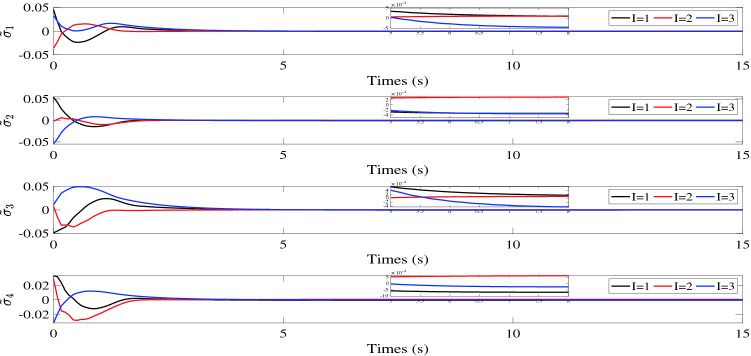
Attitude tracking errors ${\tilde{\sigma }_i}(t).$

**Figure 3:**
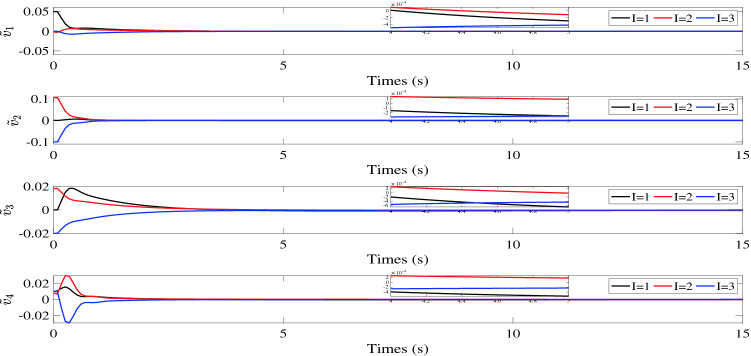
Estimation errors $${\tilde{v}_i}(t).$$

**Figure 4:**
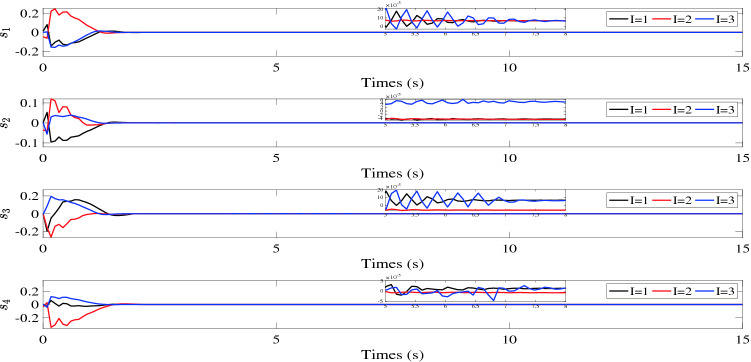
Auxiliary variable $${s_i}(t).$$

**Figure 5:**
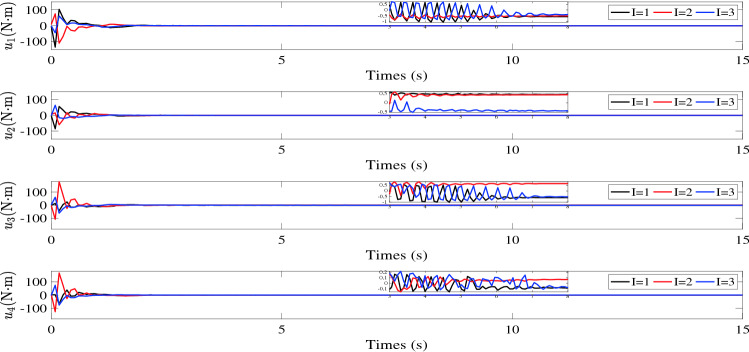
Control torques $${u_i}(t).$$

${\tilde{\sigma }_i}(t)$, ${\tilde{v}_i}(t)$ and ${s_i}(t)$ are illustrated in Figs. 2, 3 and 4, respectively. Figure 5 shows the control torques of the follower spacecraft. The attitude tracking errors ${\tilde{\sigma }_i}(t)$, estimation errors ${\tilde{v}_i}(t)$ and auxiliary variable ${s_i}(t)$ can converge to region $\left| {{{\tilde{\sigma }}_{iI}}} \right| < 7 \times {10^{ - 4}}$, $\left| {{s_{iI}}} \right| < 6 \times {10^{ - 5}}$ in 8 s and $\left| {{{\tilde{v}}_{iI}}} \right| < 6 \times {10^{ - 4}}$ in 5 s. As the desired attitude is dynamic, the control torques will never be zero, as shown in Fig. 5. After the multi-spacecraft system reaches attitude synchronization, the control torque tends to be stable. Although the fixed time control algorithm cannot consistently converge the error of the auxiliary variable to zero, as shown in Fig. 4, it can still be used to reduce the convergence time in the case of tracking the dynamic target, as the convergence time is influenced by the initial conditions in the traditional control process, which continuously change due to the dynamic reference attitude.

**Table 1:** Initial parameters of follower spacecraft.

Spacecraft *No*.*i*	Inertia matrix ${J_i}(\text {kg} {\text {m}^2})$	Initial attitude ${\sigma _i}(0)$	Initial attitude derivative ${\dot{\sigma }_i}(0)$
1	$$\left[ \begin{array}{lllllllll}{15}&{{{0}}{{.4}}}&{{{1}};}&{{{0}}{{.4}}}&{{{14}}}&{{{1}}{{.5}};}&{{1}}&{{{1}}{{.5}}}&{{{12}}} \end{array} \right]$$	$[0.0454, - 0.0230,0.0325]$	[0, 0, 0]
2	$$\left[ \begin{array}{lllllllll}{14}&{{{0}}{{.3}}}&{{{1}};}&{{{0}}{{.3}}}&{{{16}}}&{{{1}}{{.3}};}&{{1}}&{{{1}}{{.3}}}&{{{15}}} \end{array} \right]$$	$[0.0542,0.0114, - 0.0548]$	[0, 0, 0]
3	$$\left[ \begin{array}{lllllllll}{18}&{{{0}}{{.5}}}&{{{2}};}&{{{0}}{{.5}}}&{{{13}}}&{{{1}}{{.6}};}&2&{{{1}}{{.6}}}&{{{14}}} \end{array} \right]$$	$[ - 0.0486,{{0}}{{.0205,0}}{{.0110}}]$	[0, 0, 0]
4	$$\left[ \begin{array}{lllllllll} {16}&{{{0}}{{.6}}}&{1;}&{{{0}}{{.6}}}&{{{15}}}&{{{1}}{{.4}};}&1&{{{1}}{{.4}}}&{{{17}}} \end{array} \right]$$	$[0.0335,0.0414, - 0.0322]$	[0, 0, 0]

## Conclusions

This paper studies the problem of distributed coordinated control of spacecraft attitude tracking under time-varying communication delays, where the communication topology is described as a directed graph and the reference attitude is dynamic. Since the reference attitude information is available to only some of the following spacecraft, a distributed estimator that uses neighbor information under communication delay is proposed to estimate the reference attitude information. An auxiliary variable is designed based on the estimated information. By means of the fixed-time control method, a fixed-time control algorithm is proposed that achieves convergence of the auxiliary variable with fixed time, and the convergence time is independent of the initial state. This means that the influence of the constantly changing initial conditions on the system is reduced. By using the Lyapunov–Krasovskii functional approach and input-to-state stability theory, the proposed control algorithms guarantee that the attitude of each following spacecraft can be asymptotically synchronized with the dynamic attitude of the leader spacecraft.

## Data Availability

All relevant data are in the manuscript and the relevant literature cited in the manuscript.

## References

[1] Zhang K, Demetriou MA (2015). Adaptation and optimization of the synchronization gains in the adaptive spacecraft attitude synchronization. Aerosp. Sci. Technol..

[2] Ma C, Zeng Q, Zhao X (2015). Distributed adaptive attitude synchronization for spacecraft formation flying with sampled-data information flows. J. Franklin Inst..

[3] Hong, Y., Hu, J., & Gao, L. *Tracking Control for Multi-agent Consensus with an Active Leader and Variable Topology*. (Automatica-Oxford, 2006).

[4] Du H, Li S, Qiao C (2013). Decentralized slidingmode control for attitude synchronization in spacecraft formation. Int. J. Robust Nonlinear Control.

[5] Zou AM, Kumar K, Hou Z (2012). Attitude coordination control for a group of spacecraft without velocity measurements. Control Syst. Technol. IEEE Trans..

[6] Liang H, Sun Z, Wang J (2013). Finite-time attitude synchronization controllers design for spacecraft formations via behavior-based approach. Proc. Inst. Mech. Eng. Part G J. Aerosp. Eng..

[7] Zou AM, Kumar K (2012). Distributed attitude coordination control for spacecraft formation flying. IEEE Trans. Aerosp. Electron. Syst..

[8] Polyakov A (2012). Nonlinear feedback design for fixedtime stabilization of linear control systems. IEEE Trans. Autom. Control.

[9] Shi XN, Zhou D (2020). Adaptive fault-tolerant attitude tracking control of rigid spacecraft on lie group with fixed-time convergence. Asian J. Control.

[10] Ton C, Petersen C (2018). Continuous fixed-time sliding mode control for spacecraft with flexible appendages. IFAC-PapersOnLine.

[11] Gao J, Cai Y (2015). Fixed-time control for spacecraft attitude tracking based on quaternion. Acta Astronaut..

[12] Abdessameud A, Tayebi A (2012). Attitude synchronization of multiple rigid bodies with communication delays. IEEE Trans. Autom. Control.

[13] Wang HL (2014). Consensus of networked mechanical systems with communication delays: A unified framework. IEEE Trans. Autom. Control.

[14] Guo YH, Lu PL, Liu X (2015). Attitude coordination for spacecraft formation with multiple communication delays. Chin. J. Aeronaut..

[15] Zhou JK, Ma GF, Hu QL (2012). Delay depending decentralized adaptive attitude synchronization tracking control of spacecraft formation-sciencedirect. Chin. J. Aeronaut..

[16] Zhao L, Jia YM (2014). Decentralized adaptive attitude synchronization control for spacecraft formation using nonsingular fast terminal sliding mode. Nonlinear Dyn..

[17] Zhai DH, Xia YQ (2017). Adaptive control of semiautonomous teleoperation system with asymmetric time-varying delays and input uncertainties. IEEE Trans. Cybern..

[18] Meng ZY, Ren W (2010). Distributed finite-time attitude containment control for multiple rigid bodies. Automatica.

[19] Ren, W., & Cao, Y. Distributed coordination of multi-agent networks. *Commun. Control Eng.* (2010).

[20] Khalil, H. K. *Nonlinear Systems*, 3rd ed. (2002).

[21] Zuo Z, Lin T (2014). A new class of finite-time nonlinear consensus protocols for multi-agent systems. Int. J. Control.

[22] Wang D, Zhang N, Wang JK, Wang W (2017). Cooperative containment control of multiagent systems based on follower observers with time delay. IEEE Trans. Syst. Man Cybern. Syst..

[23] Gahinet P, Apkarian P (1994). A linear matrix inequality approach to H$$\infty$$ control. Int. J. Robust Nonlinear Control.

[24] Liu W, Huang J (2017). Adaptive leader-following consensus for a class of higher-order nonlinear multiagent systems with directed switching networks. Automatica.

[25] Cai H, Huang J (2014). Leader-following consensus of multiple uncertain Euler–Lagrange systems under switching network topology. Int. J. Gen. Syst..

[26] Huang WJ, Li C, Sun Y (2019). Distributed coordinated attitude tracking control for spacecraft formation with communication delays. ISA Trans..

[27] Polyak BT, Khlebnikov MV, Shcherbakov PS (2021). Linear matrix inequalities in control systems with uncertainty. Autom. Remote Control..

[28] Last E (1994). Linear matrix inequalities in system and control theory. Proc. IEEE.

